# Application of 3D printed model for planning the endoscopic endonasal transsphenoidal surgery

**DOI:** 10.1038/s41598-021-84779-5

**Published:** 2021-03-05

**Authors:** Xing Huang, Ni Fan, Hai-jun Wang, Yan Zhou, Xudong Li, Xiao-Bing Jiang

**Affiliations:** 1 Department of Neurosurgery, Union Hospital, Tongji Medical College, Huazhong University of Science and Technology, Wuhan, 430022 China; 2 Department of Gynaecological Oncology, Hubei Cancer Hospital, Wuhan, 430079 Hubei China

**Keywords:** Cancer, Surgical oncology

## Abstract

The application of 3D printing in planning endoscopic endonasal transsphenoidal surgery is illustrated based on the analysis of patients with intracranial skull base diseases who received treatment in our department. Cranial computed tomography/magnetic resonance imaging data are attained preoperatively, and three-dimensional reconstruction is performed using MIMICS (Materialise, Leuven, Belgium). Models of intracranial skull base diseases are printed using a 3D printer before surgery. The models clearly demonstrate the morphologies of the intracranial skull base diseases and the spatial relationship with adjacent large vessels and bones. The printing time of each model is 12.52–15.32 h, and the cost ranges from 900 to 1500 RMB. The operative approach was planned in vitro, and patients recovered postoperatively well without severe complications or death. In a questionnaire about the application of 3D printing, experienced neurosurgeons achieved scores of 7.8–8.8 out of 10, while unexperienced neurosurgeons achieved scores of 9.2–9.8. Resection of intracranial skull base lesions is demonstrated to be well assisted by 3D printing technique, which has great potential in disclosing adjacent anatomical relationships and providing the required help to clinical doctors in preoperative planning.

## Introduction

Success and quality of skull base surgeries usually depend on the surgeons’ experience, anatomical knowledge, and ability to imagine the 3D space. In order to achieve relatively good surgical outcomes, multiple surgical simulation trainings are necessary^1^. However, with the emergence of precision medicine, neurosurgery is developing in the direction of precision and minimal invasion. The revolutionary 3D printing technique provides guidance for precision medicine and shows great developmental potential in the clinical application^2–6^.

3D printing, which is also called additive manufacturing or rapid prototyping technology^7^, is based on model data gained from rapid image segmentation, image registration, and 3D reconstruction of imported 2D computed tomography (CT)/magnetic resonance imaging (MRI) data by a 3D visualization software. After acquisition of 3D STL (stereolithography) image files suitable for printing, physical models are printed in 1:1 scale using 3D printing equipment, which restores the intracranial anatomical structure for realistic visualization. Based on the 3D printed models, doctors can preoperatively plan the operative approach, get direct navigation for critical steps of the surgeries by adjusting the 3D printed models and locating them in the best anatomical locations, and eventually complete the surgeries successfully^8,9^. This article summarizes the evaluation of the application of 3D printing technique for the preoperative planning of endoscopic endonasal transsphenoidal surgeries performed in our department.

## Materials and methods

### Patient information

We analyzed the data of four patients who received treatment in our department between April and December 2019. Clinical data, including gender, age, symptoms, surgical approach, tumor location and size, and pathological diagnosis, were recorded. The protocol for this study was reviewed and approved by the Ethics Committee of Tongji Medical College, Huazhong University of Science and Technology, and written informed consent was obtained from each patient, in accordance with the principles expressed in the Declaration of Helsinki.

### Image acquisition

A Siemens dual-source 128-slice spiral CT scanner (Siemens Healthcare, Forchheim, Germany) was used for continuous axial tomography of the skull with 0.625-mm slice thickness and 0.35-mm in-plane resolution, scanning parameters of 120 kV and 205.50 mAs, and a scanning matrix of 512 × 512 in size. MRI images of the corresponding region were acquired using a 1.5-T unit (Siemens Sonata; Siemens, Medical Solutions, Erlangen, Germany). Postcontrast T1-weighted axial images (TR, 512 ms; TE, 13 ms; 1-mm-thick slices) were merged with CT images for a better bone-soft tissue contrast. Imaging data, including CT data of skull and cervical vertebrae, MRI of the tumors, and CT angiography (CTA) of the internal carotid artery, were obtained, saved in DICOM (Digital Imaging and Communications in Medicine) format, and exported.

### 3D printing processing

DICOM data from patients’ high-resolution (0.625-mm-thick slices) CT and CTA scans of the skull bone and cranial arteries were imported into the computer-aided design (CAD) software (Materialise, Leuven, Belgium). Therein, bony and artery anatomies were segmented from surrounding structures according to a gray scale, and tissues with a certain level of gray were converted into 3D images through the algorithm of the software. In contrast-agent CT imaging, the blood vessels’ density value is similar to that of the bone, which facilitates the reconstruction of the shape of skull and blood vessels. In the following, we focused on the anatomical structure of the sellar region and its surroundings. MRI data from T1-weighted axial images were used for the 3D reconstructing of tumors. Based on their anatomical relationships, different parts were matched in the same software to restore a model file that coincides with the characteristics of the patient’s lesions. Different colors were used to mark the different modules. The final fused 3D structure was stored as a stereo model file in the STL format and imported into the 3D printer for printing. In this study, the prototype was obtained by a PolyJet process for additive manufacturing on an Objet350 Connex printer (Stratasys, Eden Prairie, MN). The 1:1 realistic model was constructed from acrylonitrile butadiene styrene (ABS). The printer printed the models in layers coincident with the requirements and according to the structural information contained in the STL file.

### Model evaluation and application

The main structures of the lesion models printed by the 3D printer were evaluated by five experienced neurosurgeons with at least 5 years working experience in endoscopic surgery, as well as five unexperienced neurosurgeons. We confirmed the lesions’ locations, directions, sizes, as well as their positional relationship with the main adjacent arteries. Furthermore, different surgical approaches were tested on the models in order to elaborate the best operative approach. The models printed by the 3D printer were observed and measured at various angles. Lastly, the neurosurgeons were asked to complete a postoperative questionnaire consisting of seven questions, which were defined according to Ryan et al.^10^, to evaluate their satisfaction with the 3D printed model. Differences between the two groups were analyzed by the chi-squared test. All statistical analyses were carried out by SPSS software version 13.0 (Statistical Package for the Social Sciences; SPSS Inc., Chicago, IL, USA).

### Ethical approval

The protocol for this study was reviewed and approved by the Ethics Committee of Tongji Medical College, Huazhong University of Science and Technology (Huazhong, Wuhan, China).

## Results

### 3D printing processing data

For every patient, the lesion model restored the anatomical relationship with adjacent vessels and bones. Furthermore, lesions, vessels, and skull were marked with different colors for an improved observation and perspective. Acrylate resin was used as the 3D-printed material, and the total time, including designing and printing time, ranged from 12.52 to 15.32 h. All the neurosurgeons were satisfied with the models, and the cost of the 3D printed models was 900–1500 yuan. Detailed information is given in Table 1.

**Table 1:** The datas of 3D printing processing.

Patient	Material	Process time (hours)	Satisfaction	Cost (CNY)
1	Acrylate resin	15.32	Yes	1500
2	Acrylate resin	12.78	Yes	1000
3	Acrylate resin	13.92	Yes	1200
4	Acrylate resin	12.52	Yes	900

*CNY* Chinese yuan.

### Evaluation of 3D printed models

**Figure 1:**
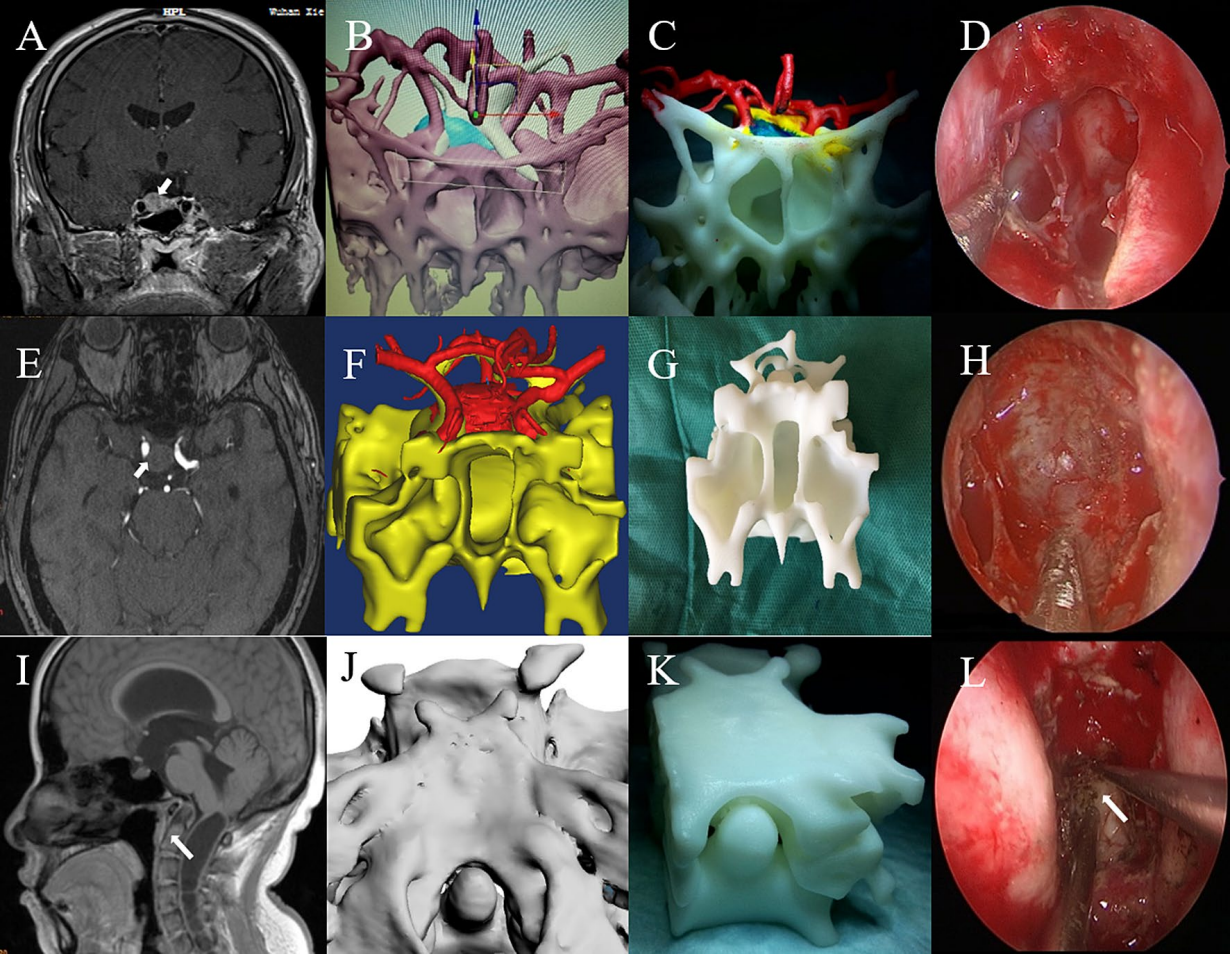
MRI, 3D printing data, and photographs of three patients. **(A/E/I)** MRI images of two patients with pituitary and one with odontoid deformity. The nidus is marked by a white arrow. **(B/F/J)** 3D reconstruction of each nidus in the software of MIMICS. **(C/G/K)** 3D printed models for each patient. **(D/H/L)** Intraoperative photographs of each patient.

The entity of the lesion allowed the direct observation of the lesion’s location, size, and anatomical relationship with adjacent tissue. It also reflected critical anatomical perspectives, which corresponded to intraoperative findings (Fig. 1). The models were applied to realistically evaluate the operative approach and the anatomical position relative to the adjacent structure. Although experienced neurosurgeons achieved lower average scores in seven questions about the application of 3D printing than unexperienced neurosurgeons, no significant difference in the distribution of the scores between these two groups of neurosurgeons was found. Detailed information is shown in Table 2.

**Table 2:** Average scores of the questionnaire used by experienced and unexperienced neurosurgeons to evaluate the 3D printed model (scores range from 1 to 10).

Questions	Group		
	Experienced neurosurgeons	Unexperienced neurosurgeons	P value
1. Is the simulator clinically applicable?	7.8	9.2	0.0578
2. Did the simulator improve your understanding of the tumor’s relationship to the parent artery?	8.4	9.4	> 0.9999
3. Did the simulator improve your understanding of the surgical view?	8.6	9.2	0.1967
4. Did the 3D printed model seem realistic?	8.8	9.8	0.4902
5. Was the bone drilled in a realistic manner?	8.8	9.2	0.0578
6. Did you find the simulator useful?	8.6	9.4	0.5271
7. Do you think your surgical skills would improve with practice using the simulator?	7.8	9.6	0.4902

### Clinical outcomes

Three male patients and one female patient were enrolled in this study, and their ages ranged from 24 to 55 years with an average age of 44 years. The skull base cases comprised two pituitary adenomas, one chordoma, and one odontoid deformity. Table 3 shows the detailed data. In the preoperative planning, all four patients received surgeries as their operative approaches, and all the tumors were resected. All patients recovered postoperatively well, and no severe complications or death occurred.

**Table 3:** Data of patients who received resection of intracranial skull base lesions with the assistance of 3D printing technique.

Patient	Gender	Age (yr)	Symptom	Lesion location	Lesion size (mm)			Surgical approach	Pathologic diagnosis
					L	W	H		
1	M	47	Visual decline	Sella turcica	53	44	33	EETA	PA
2	M	55	No symptom	Sella turcica	11	12	11	EETA	PA
3	F	23	Walking unsteadily	Odontoid	10	10	13	EETA	Odontoid deformity
4	M	42	Hoarseness	Clivus	57	32	35	EETA	Chordoma

*M* male, *F* female, *yr* year, *L* length, *W* width, *H* height, *EETA* endoscopic endonasal transsphenoidal approach, *PA* pituitary adenoma.

## Discussion

3D printing technique is receiving worldwide more and more attention in assisting surgeries in medicine and, especially, in neurosurgery. Due to the complicated 3D anatomical structure of the central nervous system and individual differences among patients, precise resection of lesions in the brain parenchyma still presents a challenge for neurosurgeons. With the rise of the 3D printing technique, not only 2D pictures but also anatomical structures that meet the requirements can be converted into 3D structures. This presents a tremendous support for surgeries that are based on anatomical knowledge. Souzaki et al. printed 3D neurocytoma kidney models based on CT imaging for preoperative simulations of laparoscopic adrenal gland resection surgeries on children and infants, which confirmed the importance of the simulations for the familiarization with the patient’s anatomical structure and the planning of the operation^11^. Kawaguchi et al. used the 3D printing technique to construct surgical navigation models in order to assist the treatment of cervical diseases by inserting screws and Magerl nails in the pedicle with satisfying surgical outcomes^12^. Lu et al. used 3D-printed guide plates to insert nails into the atlantoaxial area, the once so-called “forbidden zone”. First, they constructed centrum models and guide plates. After testing the accuracy of the guide plates on the models, they performed the nail-insertion surgery with the assistance of the guide plates and achieved good surgical outcomes^13^. Spottiswoode et al. used 3D printing technique to print brain tissue models of patients. They preoperatively designed the optimal operative approach, located the lesion and the adjacent functional zone accurately according to superficial brain sulci and gyri, confirmed the resection extension, and increased the accuracy and safety of the surgery process^14^. Patients recovered well postoperatively.

In this study, we explored the use of the 3D printing technique for skull base diseases, such as pituitary adenoma, odontoid deformity, and chordoma. The results are summarized below.

### Practical value of the 3D printing technique

In view of increasingly less opportunities to practice surgical techniques, training and preoperative planning with surgical simulators are becoming more and more important for neurosurgeons, especially for the operation of large and complex skull base tumors. Although the rise of neuronavigation solved the problem of locating cranial lesions to a certain degree, the navigation method requires a screen, which entails the inherent limitation of displaying 3D anatomical structures on 2D planes. Furthermore, the navigation system requires patients to register preoperatively. During the operation, localization of tumors depends on signal acquisition by the navigation equipment for spatial orientation and displaying the results on the screen. This method depends on the emission, acquisition, and transition of signals, increases the complexity of the operation, and decreases the accuracy of localization during the operative time. Complicated operations and high costs also limit the promotion of the neuronavigation technique. In addition, although the existing 3D imaging technology and virtual reality technology can help doctors to a great extent in preoperative planning, preoperative practice is indispensable for younger or older doctors who attempt difficult operations. In case 3 of Table 1, we had no experience in removing the odontoid process by the transsphenoidal approach, and we could not get sufficient information from the virtual image. Therefore, we reconstructed the structure of the disease by 3D imaging technology before operation, which was necessary to some extent. Model and data were stored to improve our future training. In our questionnaire survey, both groups of experienced and unexperienced neurosurgeons achieved high average scores (> 7), demonstrating general clinical practicability of 3D printing. The average score of the experienced neurosurgeons was lower than that of the unexperienced neurosurgeons, which was mainly ascribed to the experienced doctors’ accumulated clinical experience and thus lower need for assistance. However, both groups generally rated the application of 3D printing as positive, as indicated by the small variability in the scores among different questions. Among all questions, the lowest score was related to clinical value prospects, as the higher cost was considered to limit the clinical application. To solve this problem, we printed out the key structures instead of the whole skull and its appendages. Table 2 shows that the cost per patient ranges from 900 to 1500 RMB, which is much lower than that of other reports. However, costs are additionally saved by printing out key structures, which decreased the printing time of 12.52–15.32 h. Therefore, in clinical practice, the shortcomings of 3D printing technology should be overcome to fully exploit its advantages and demonstrate its real value.

### Localization of cranial lesions and planning of operative approaches

For each of the four patients, we reconstructed the anatomical structure of the disease and demonstrated the relationship between lesion and adjacent bone and vessels. In endoscopic transsphenoidal surgeries, natural canals of the human body are used for removing midline tumors by a minimally invasive surgery approach. However, due to the complicated structure of the nasal cavity and the large amount of important nerves and vessels in the midline zone, the locations of important structures on the skull base, such as internal carotid, optic nerve, and tumor orientation, need to be repeatedly confirmed during operation. Although most tumors can be resected with the assistance of the intraoperative navigation system, intraoperative localization is still based on 2D identification. When the structure of the nasal cavity is complicated or when the tumor is hidden, surgeons need to judge according to their own experience. Due to differences in the experience of different doctors and occasionally complicated operative situations, the outcomes of surgeries vary and may not even be satisfying. The application of the 3D printing technique in neurosurgical models helps doctors to directly localize and get information about adjacent vessels and nerves. It provides personalized and accurate guidance for surgical steps. For our four patients, we successively applied 3D printing technique in skull base surgeries, and we performed preoperatively a realistic simulation of the endoscopic endonasal transsphenoidal approach based on the reconstructed models.

For the patients of Table 1 whose reconstruction is shown in Fig. 1, the anatomical structure of the sphenoid sinus and the vascular structure of the intracranial sellar region could be clearly evaluated by the three-dimensional model of pituitary adenoma in two patients. From the model, we could roughly determine the locations of the tumors, which were consistent with the intraoperative findings. At the same time, the direction of the internal carotid artery could be identified by the help of the model, which is very important for transnasal sphenoidal surgery. We initially planned our surgical approach on the model, and the operations of two patients were successful without cerebrospinal fluid rhinorrhea.

Due to the abnormal protrusion of the odontoid of patients with basilar invagination, the ventral regions of the medulla oblongata and cervical cord are compressed and lead to clinical symptoms^15^. With the rapid rise of the endoscope technique, endoscopic transnasal-sphenoidal resection of odontoid lesions has become feasible^16^. Due to the complicated anatomical structure and biomechanical characteristics of the craniocervical junction, the surgical treatment is very challenging. In our group, we had no practical experience with this kind of surgery before, so we urgently needed this model to train our surgical skills. More importantly, unlike conventional transnasal sphenoidal surgery, the transnasal odontoid approach has a deeper location and requires higher intraoperative localization, and these problems could be solved by the 3D model. We rehearsed the surgical approach repeatedly on the model to remove the lesions to the greatest extent. The 3D model was consistent with the intraoperative findings.

### Limitations

Designing accurate models requires images that strictly meet the demands. All our patients performed scanning to obtain strictly required data. In order to get more information from all sides, patients were usually required to complete at least two examinations. Differences between the two examinations in posture and scanning time can cause a certain error. The parts extracted from different examinations need to be assembled according to their anatomical relationship, which requires the person who performs the reconstruction to have a certain anatomical knowledge. Even after assembling, a drift in the position may exist among different modules. However, the models provided very good guidance for the surgeries of four patients in this study. Further experiments need to confirm whether the error of the model is in the range of acceptance and whether a position drift exists in the critical anatomical parts. After constructing a model, it was printed with the 3D printer. However, due to the layer-by-layer accumulation principle, it usually takes several hours to print a simple model, which impedes an application of the 3D printing technique in emergency surgeries. Moreover, an application of the 3D printing technique is limited by the choice of material, as well as the cost of the models^17,18^.

In conclusion, 3D printing is still in its initial stage of clinical application. Its possibilities of personalized model localization and demonstration of special anatomical relationships can support the establishment of operation plans, leading to a high relevance of promoting and developing the 3D printing technique for medical applications.

## References

[1] Kshettry VR, Mullin JP, Schlenk R, Recinos PF, Benzel EC (2014). The role of laboratory dissection training in neurosurgical residency: Results of a national survey. World Neurosurg..

[2] Tan ET, Ling JM, Dinesh SK (2016). The feasibility of producing patient-specific acrylic cranioplasty implants with a low-cost 3D printer. J. Neurosurg..

[3] Huang, X. *et al*. A small 3D-printing model of macroadenomas for endoscopic endonasal surgery. **22**, 46–53, 10.1007/s11102-018-0927-x (2019). 10.1007/s11102-018-0927-x PMC6373287 30506234

[4] Essayed WI (2018). 3D printing and intraoperative neuronavigation tailoring for skull base reconstruction after extended endoscopic endonasal surgery: Proof of concept. J. Neurosurg..

[5] Gao F, Wang Q, Liu C, Xiong B, Luo T (2017). Individualized 3D printed model-assisted posterior screw fixation for the treatment of craniovertebral junction abnormality: A retrospective study. J. Neurosurg. Spine.

[6] Tai BL (2015). Development of a 3D-printed external ventricular drain placement simulator: Technical note. J. Neurosurg..

[7] Tack P, Victor J, Gemmel P, Annemans L (2016). 3D-printing techniques in a medical setting: A systematic literature review. Biomed. Eng. Online.

[8] George E, Liacouras P, Rybicki FJ, Mitsouras D (2017). Measuring and establishing the accuracy and reproducibility of 3D printed medical models. Radiographics.

[9] Lin J (2018). Using three-dimensional printing to create individualized cranial nerve models for skull base tumor surgery. World Neurosurg..

[10] Ryan JR, Almefty KK, Nakaji P, Frakes DH (2016). Cerebral aneurysm clipping surgery simulation using patient-specific 3D printing and silicone casting. World Neurosurg..

[11] Souzaki R (2015). Preoperative surgical simulation of laparoscopic adrenalectomy for neuroblastoma using a three-dimensional printed model based on preoperative CT images. J. Pediatr. Surg..

[12] Kawaguchi Y (2012). Development of a new technique for pedicle screw and Magerl screw insertion using a 3-dimensional image guide. Spine.

[13] Lu S (2009). A novel computer-assisted drill guide template for placement of C2 laminar screws. Eur. Spine J..

[14] Spottiswoode BS (2013). Preoperative three-dimensional model creation of magnetic resonance brain images as a tool to assist neurosurgical planning. Stereotact. Funct. Neurosurg..

[15] Wang, M. Y. Cervical crossing laminar screws: Early clinical results and complications. *Neurosurgery* **61**, 311–315 (discussion 315–316), 10.1227/01.neu.0000303987.49870.7b (2007). 10.1227/01.neu.0000303987.49870.7b 18091245

[16] Deopujari CE, Karmarkar VS, Shah NJ (2014). Endoscopic approaches to the craniovertebral junction and odontoid process. World Neurosurg..

[17] Pucci JU, Christophe BR, Sisti JA, Connolly ES (2017). Three-dimensional printing: Technologies, applications, and limitations in neurosurgery. Biotechnol. Adv..

[18] Wilcox B, Mobbs RJ, Wu AM, Phan K (2017). Systematic review of 3D printing in spinal surgery: The current state of play. J. Spine Surg. (Hong Kong).

